# Fiber-Array-Based Raman Hyperspectral Imaging for Simultaneous, Chemically-Selective Monitoring of Particle Size and Shape of Active Ingredients in Analgesic Tablets

**DOI:** 10.3390/molecules24234381

**Published:** 2019-11-30

**Authors:** Timea Frosch, Elisabeth Wyrwich, Di Yan, Juergen Popp, Torsten Frosch

**Affiliations:** 1 Leibniz Institute of Photonic Technology, 07745 Jena, Germany; timea.frosch@uni-jena.de (T.F.); elisabeth.wyrwich@leibniz-ipht.de (E.W.); di.yan@leibniz-ipht.de (D.Y.); juergen.popp@ipht-jena.de (J.P.); 2 Institute of Physical Chemistry, Friedrich Schiller University, 07743 Jena, Germany; 3 Abbe Centre of Photonics, Friedrich Schiller University, 07745 code Jena, Germany

**Keywords:** Raman spectroscopy, fiber-array, hyperspectral imaging, chemical imaging, fiber sensing, acetylsalicylic acid, acetaminophen, caffeine, particle size, particle shape, API distribution, pharmaceutical spectroscopy

## Abstract

The particle shape, size and distribution of active pharmaceutical ingredients (API) are relevant quality indicators of pharmaceutical tablets due to their high impact on the manufacturing process. Furthermore, the bioavailability of the APIs from the dosage form depends largely on these characteristics. Routinely, particle size and shape are only analyzed in the powder form, without regard to the effect of the formulation procedure on the particle characteristics. The monitoring of these parameters improves the understanding of the process; therefore, higher quality and better control over the biopharmaceutical profile can be ensured. A new fiber-array-based Raman hyperspectral imaging technique is presented for direct simultaneous in-situ monitoring of three different active pharmaceutical ingredients- acetylsalicylic acid, acetaminophen and caffeine- in analgesic tablets. This novel method enables a chemically selective, noninvasive assessment of the distribution of the active ingredients down to 1 µm spatial resolution. The occurrence of spherical and needle-like particles, as well as agglomerations and the respective particle size ranges, were rapidly determined for two commercially available analgesic tablet types. Subtle differences were observed in comparison between these two tablets. Higher amounts of acetaminophen were visible, more needle-shaped and bigger acetylsalicylic acid particles, and a higher incidence of bigger agglomerations were found in one of the analgesic tablets.

## 1. Introduction

The primary goal of the pharmaceutical industry is the achievement of an economic production of high-quality products with efficient and safe therapeutic effects for patients who rely on medical treatment. The particle size and shape are quality indicators to a great extent due to their high influence on the flow, mixing, and compaction properties, and thus on the production processes and the distribution of the active pharmaceutical ingredients (APIs) in the final product [1] Larger, more spherical particles have better flow properties than smaller or high aspect ratio particles (particle length >> particle width), ensuring a better dose-accuracy. Mixing of active agents and excipients with different shapes and sizes can cause de-mixing phenomena. Needle-like particles, such as the ones of acetylsalicylic acid, can associate better. They can form a network, in which smaller particles like the ones of caffeine can eventually be embedded, enabling a more stable mixture of these two APIs. A homogenous mixture is challenging to achieve when the particle sizes of the acetylsalicylic acid–caffeine complexes are not in the same size range as the acetaminophen particles. Hence, the homogeneous distribution of the three APIs in a volume-dosed product would not be ensured. The dosage form, such as the tablet, is the form that comes into direct physiological contact with the patient. The knowledge regarding the ingredient parameters in the end product is of high relevance, because the size, shape, and distribution of the API particles also have direct biopharmaceutical, (patho-)physiological and pharmacological consequences. Small particles are solved quickly, and the absorption of the active ingredient takes place in a short time after ingestion. Thus, the bioavailability is enhanced, but eventually the toxicity is too. Bigger, needle-like particles of acetylsalicylic acid irritate the stomach mucosa and facilitate the occurrence of gastric ulcer and gastrointestinal bleeding [2]. However, the high absorption rate of small particles in high doses promotes interactions with other medications at the plasma protein binding level [3]. A homogenous distribution and hence simultaneous dissolution of the APIs in the tablet is desired for its enhanced pharmacological effect of the combined medicinal products. In our study the synergistic analgesic effect of acetylsalicylic acid and acetaminophen combined with caffeine [4] is targeted in the two commercial tablets, Thomapyrin Intensiv^®^ (T) and Neuranidal^®^ N (N).

The monitoring of the particle shape and size of the different ingredients simultaneously and in-situ after every process-step would be beneficial for better control of the quality. This would also help to fulfill the requirements of the authorities, for example on uniformity of content and dosage units [5]. Determining the particle size, shape and distribution of the APIs in the product would help to ensure the safety and therapeutic effects of the tablets.

As for now, particle size and shape are routinely measured in raw powder material. Numerous methods are available. Sieve analysis and sedimentation analysis [6] are used for the determination of the amounts of the different particle types, whereas laser diffraction, coulter counter or photonic-correlation-spectroscopy [7] and various techniques of light-, electro- and atomic force microscopy are applied as counting-methods of the different particle sizes [6]. None of these methods can chemically differentiate the ingredients of the powder mixture and they are not suitable for chemical selective characterization of the particles in a tablet. Furthermore, due to high shear forces during powder homogenization, the particles can reduce their dimensions and round-shaped particles become dominant. On the other hand, very small particles tend to agglomerate, due to their enlarged active surface. At the compaction step, larger, needle shaped particles can break, whereas small particles can be packed together. Therefore, in the tablet, the particle size and shape of an API does not necessarily equal the properties of the API in the powder state anymore.

To receive chemical and spatial information at once, chemical imaging techniques have been developed, combining spectroscopy with spatially resolved information. The goal is to generate data cubes that contain the intensity values in dependence of the spectral and spatial variables I(x,y,λ). By choosing characteristic spectral peaks, specific images for various substances in the sample can be created. To extract the relevant information, multivariate chemometric methods can be applied [8,9,10]. Several chemical imaging techniques have been developed, based on mass spectrometric imaging (MSI) [11,12], terahertz pulse imaging (TPI) [13,14,15] and vibrational spectroscopic imaging techniques [16,17] such as near infrared (NIR)-imaging, attenuated total reflection Fourier-transform infrared imaging (ATR-FT-IR) [18,19,20,21,22] and Raman spectroscopic imaging [11,23,24,25,26,27,28]. Raman spectroscopy is a powerful analytical technique [29,30,31,32,33,34,35,36,37] that can also be applied in the pharmaceutical industry [17,38] It requires little or no sample preparation [39,40,41,42,43,44], is performed in a non-destructive way [45,46,47,48,49,50] and is also suitable for the chemically selective investigation of heterogeneous formulations. Pharmaceutical applications of Raman spectroscopy range from investigations regarding sample homogeneity [51], particle size [23], polymorphic forms [52], the analysis of incoming raw materials [53], counterfeit drugs [12,28], and even through containers and bags [54] to analyze pharmaceutical formulations [23,51,55,56].

The advantage of Raman imaging in comparison to mass spectrometry (MS)-based chemical imaging techniques is that it can easily be applied as a non-destructive in-process control tool. Compared with near infrared and mid-infrared spectroscopic techniques, Raman spectroscopy provides better spectral resolution [57], which helps to distinguish substances of multicomponent formulations in a highly specific way [58]. In addition, in comparison to TPI and ATR-FT-IR-imaging techniques, Raman chemical imaging provides a better spatial resolution down to approx. 1 µm [59], whereas the average resolution of the TPI systems is about 200 μm [14], and the best performance of micro-ATR-FT-IR imaging using a germanium ATR crystal is around 2–4 µm [16]. Better spatial resolution is important to resolve particle sizes and shapes within pharmaceutical dosage forms.

Raman spectroscopy is mostly applied as a mapping technique [60], using a motorized stage for precise sample movement. The most common mapping approaches for the characterization of pharmaceutical formulations are point mapping [61] or line scanning [10,62] modes. Mapping procedures are time-consuming and can last from several hours up to over a whole day, depending on the area, acquisition time/spot and step-size applied. In this work, we present the application of wide-field Raman imaging, based on a fiber-array bundle, with high spatial resolution of 1.25 µm. By use of an 8 × 8-array we acquired 64 spectra of different spots in one single acquisition. By gaining thorough chemical information across a defined sample area in a rapid way, the requirements can be met for chemically selective in-process monitoring of the size, shape and distribution of different ingredients, simultaneously, in commercially available analgesic tablets with three active ingredients.

## 2. Results and Discussion

**Figure 1:**
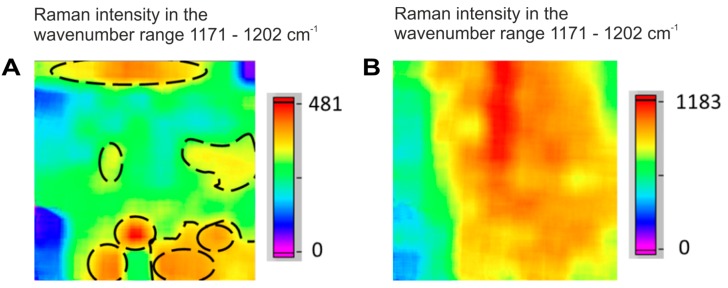
Raman hyperspectral images of acetylsalicylic acid particles in two different tablet regions. (**A**) Area with clear and distinct particle shapes and sizes (source: tablet T, Region 4). (**B**) Area with agglomerated particles (source: tablet N, Region 3).

**Figure 2:**
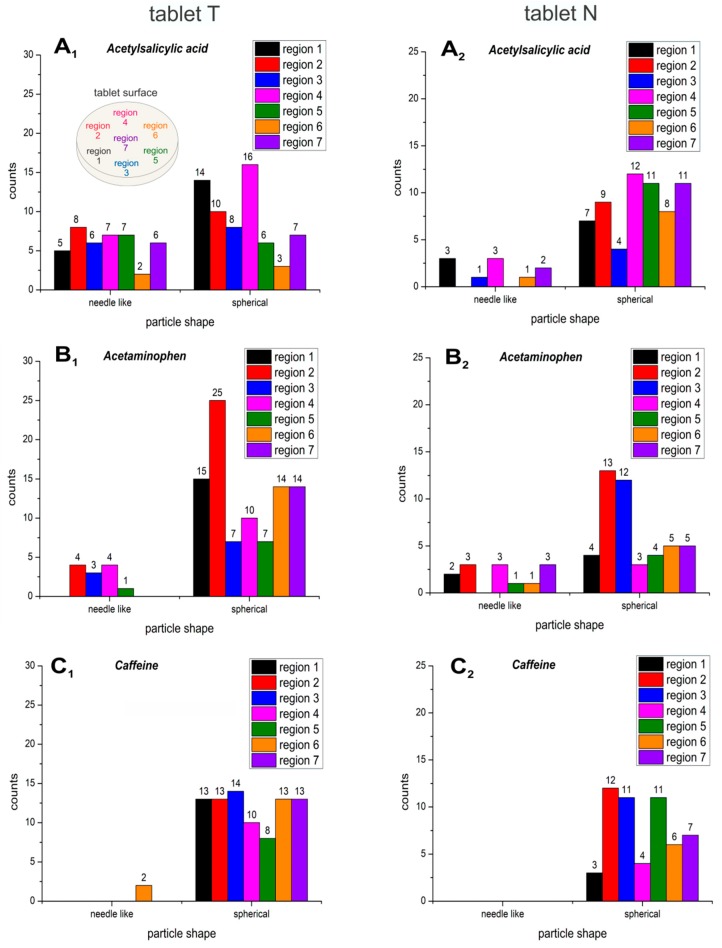
Histograms of the counted needle-like (left parts in the graphs) and spherical (right parts in the graphs) particle shapes of the active ingredients acetylsalicylic acid (**A_1_**, **A_2_**), acetaminophen (**B_1_**, **B_2_**) and caffeine (**C_1_**, **C_2_**), which were detected across seven different regions (see the sampling pattern at **A_1_**) in the two commercial tablets T (left column: **A_1_**, **B_1_**, **C_1_**) and N (right column: **A_2_**, **B_2_**, **C_2_**).

**Figure 3:**
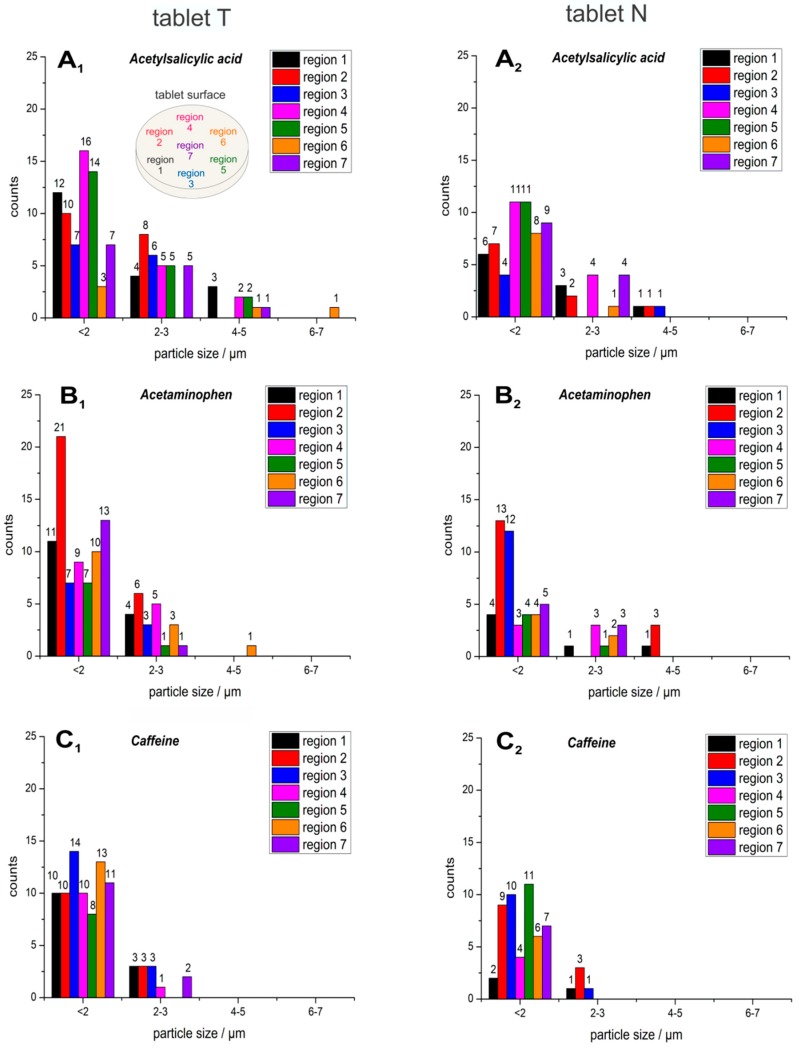
Histograms of the detected particle sizes of the active ingredients acetylsalicylic acid (**A_1_**, **A_2_**), acetaminophen (**B_1_**, **B_2_**) and caffeine (**C_1_**, **C_2_**), which were detected across seven different regions (see the sampling pattern at **A_1_**) in the commercial tablets T (left column: **A_1_**, **B_1_**, **C_1_**) and N (right column: **A_2_**, **B_2_**, **C_2_**).

**Figure 4:**
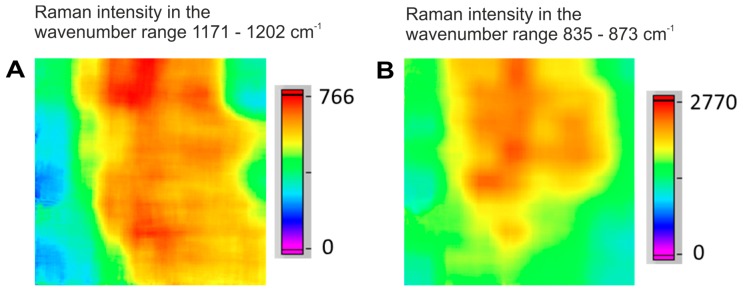
Raman hyperspectral images of agglomerated particles of (**A**) acetylsalicylic acid (source: tablet T, region 1) and (**B**) acetaminophen (source: tablet N, region 1) in two different tablet regions.

**Figure 5:**
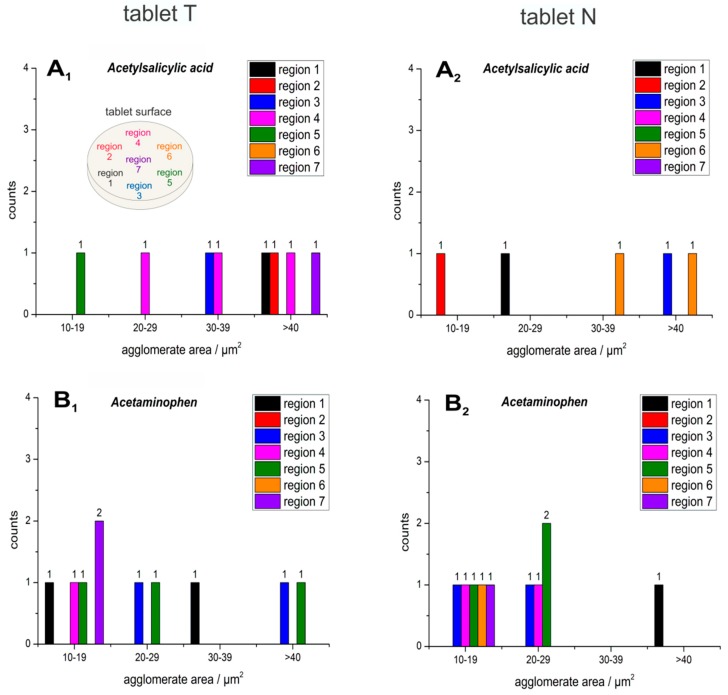
Histograms of the detected agglomerations of the API particles and their respective area sizes of acetylsalicylic acid (**A_1_**, **A_2_**), acetaminophen (**B_1_**, **B_2_**), which were detected across seven different regions (see the sampling pattern at **A_1_**) in the two tablets T (left column: **A_1_**, **B_1_**) and N (right column: **A_2_**, **B_2_**).

**Figure 6:**
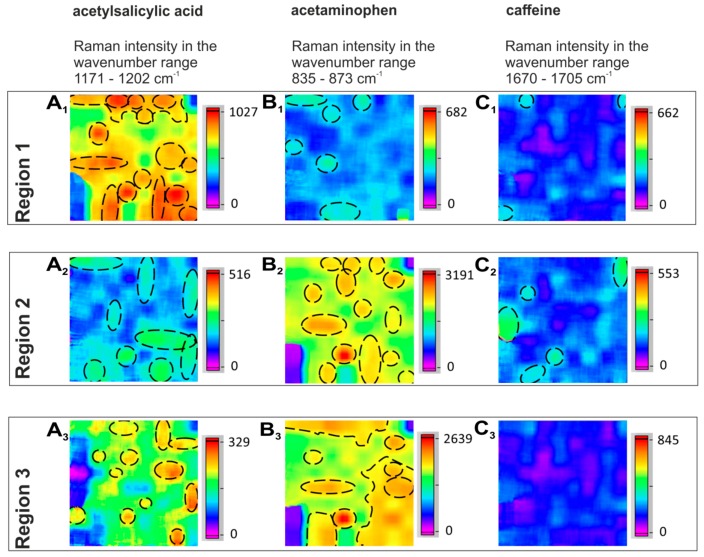
Representative Raman hyperspectral images of the distributions and particle shapes of the individual APIs, for acetylsalicylic acid (left column **A_1_**, **A_2_**, **A_3_**), acetaminophen (middle column **B_1_**, **B_2_**, **B_3_**), and caffeine (right column **C_1_**, **C_2_**, **C_3_**), in three different regions of a tablet.

Raman imaging was applied to visualize the size, shape and distribution of particles of acetylsalicylic acid, acetaminophen and caffeine chemically selective in two analgesic tablets. The Raman hyperspectral images (Figure 1, Figure 2, Figure 3, Figure 4, Figure 5 and Figure 6) are color-coded images with corresponding Raman intensity scale bars. The intensity scale bars range from purple (0) to red (maximum). At every tablet surface, hyperspectral images were acquired in seven regions distributed over the whole tablet surface (Region 7: center part; Regions 1–6: outer parts). The hyperspectral images were generated for the single APIs in every region (1–7) and the particles were counted and given as a sum value over the two imaged tablet surfaces of the two tablet types T and N as histograms (Figure 2, Figure 3 and Figure 5). Needle-like particles were defined as particles with significant differences in length and width, whereas round particles were more isotropic in their dimensions. Agglomerated particles were defined as regions with an area of at least 10 µm^2^ with a corresponding intensity of at least 2/3 of the maximum intensity (yellow to red in the intensity scale bar). The agglomeration classes were defined, based on the dimension of the covered area.

### 2.1. Particle Size and Shape

In certain sample areas (for example Region 4 of tablet T) one could clearly distinguish the size and shape of individual acetylsalicylic acid particles (Figure 1A), while in other areas (for example in tablet N, Region 3) particle agglomeration was seen (Figure 1B).

As the different parts of the same tablet were very heterogeneous in their composition, it was necessary to analyze various regions time efficiently, to gain an overview of the distribution and the particle sizes and shapes of the APIs in the formulation. Seven different areas of the two tablets T and N were analyzed for a better overview and are summarized in histograms (Figure 2 and Figure 3).

It could be observed that acetylsalicylic acid particles exist in both spherical (71.19%) and needle-like (28.81%) shapes (Figure 2A_1_,A_2_). The smaller, round forms dominated in both tablets from different manufacturers (Figure 2 and Figure 3). The particles of acetaminophen were predominantly spherical. Needle-like particles of acetaminophen were rare (15.3%) (Figure 2B_1_,B_2_). The particles of caffeine were round at all analyzed sample spots (Figure 2C_1_,C_2_). Overall, spherical particles predominate for all APIs. No inclusion of caffeine particles between the acetylsalicylic acid particles was observed, as the acetylsalicylic acid particles were fine [1] themselves, with lengths ranging between 1.25 and 7 µm (Figure 3A_1_,A_2_). It is expected that acetylsalicylic acid is well tolerated by the application of the analyzed tablets with reduced risk for gastrointestinal-mucosa-lesions. The particle size for acetaminophen ranged between 2 and 5 µm (Figure 3B_1_,B_2_), whereas caffeine particle sizes were found predominantly between 1.25 and 3 µm (Figure 3C_1_,C_2_). In total, the average particle size was 2.20 ± 1.17 µm for acetylsalicylic acid, 1.71 ± 0.68 µm for acetaminophen, and 1.90 ± 0.74 µm for caffeine in tablet T, and 1.96 ± 0.40 µm for acetylsalicylic acid, 2.36 ± 0.90 µm for acetaminophen, and 1.99 ± 0.23 µm for caffeine in tablet N.

Small particles have an enlarged surface area, enabling pronounced electrostatic and van-der-Waals interactions, and more possibilities for hydrogen bonding. Therefore, they present a high tendency for agglomeration, as can be observed for acetylsalicylic acid (Figure 4A and Figure 5A_1_,A_2_) and for acetaminophen (Figure 4B and Figure 5B_1_,B_2_).

### 2.2. Distribution of the APIs

The distribution of the APIs, acetaminophen, acetylsalicylic acid and caffeine, was analyzed in seven different areas, 10 × 10 µm^2^ each, in two different tablets. Representative Raman hyperspectral images of the individual APIs in three tablet regions are shown in Figure 6. In Region 1, acetylsalicylic acid (Figure 6A_1_) dominates and a few needle-like particles are visible. In Region 2, all three APIs can be seen (Figure 6A_2_,B_2_,C_2_). The acetylsalicylic acid particles are mostly needle like (Figure 6A_2_). Acetaminophen provides the strongest signals and shows mostly spherical particle shapes (Figure 6B_2_). Only a few spherical caffeine particles are detected (Figure 6C_2_). In Region 3, both acetylsalicylic acid and acetaminophen are present, and some acetaminophen agglomerations can be observed. In most cases, only acetaminophen or/and acetylsalicylic acid were observed in the images (e.g., Figure 6, Regions 1 and 3), because their amount was four to five times larger than the amount of caffeine and the caffeine particles were often smaller than 1 µm so that they could not be clearly resolved (e.g., Figure 6C_3_).

**Figure 7:**
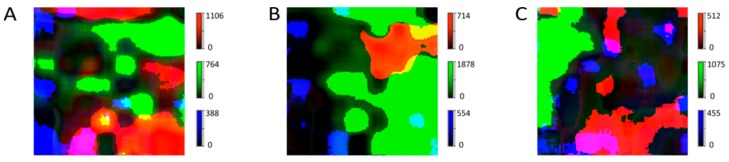
Combined distribution of acetylsalicylic acid, acetaminophen and caffeine in three different areas (**A**, **B**, **C**) of one tablet. The red-colored parts represent acetylsalicylic acid, the green-colored parts represent acetaminophen, and the blue-colored parts represent caffeine. The dark-grey areas are the excipients’ areas.

In some representative areas (e.g., T: Region 6 or N: Region 3 and Region 5), all three APIs were observed in one field of view (Figure 7A–C). The red-colored particles represent acetylsalicylic acid, the green-colored ones are acetaminophen, and the blue ones represent caffeine (Figure 7). The Raman scattering intensities of the excipients’ signals were about four to five times lower than the ones from the APIs and are presented as a grey background area without a clear particle shape. This result demonstrated the capability of fiber-array-based Raman spectroscopic imaging to visualize the API distribution, and to analyze different morphological characteristics simultaneously with high spatial resolution in just one measurement (Figure 7). This ability has high potential for in-process quality assessment.

### 2.3. Comparison of Two Analgesic Tablets

In general, very fine and round particles dominated in both tablets, including the acetylsalicylic acid particles. By comparison of the two commercial analgesic tablets, only minor differences could be recognized between T and N. First, there were about three times more needle-shaped acetylsalicylic acid particles in the T tablet (39.05%) (Figure 2A_1_) compared to the N tablet (13.89%) (Figure 2A_2_). Ten percent more spherical particles of acetaminophen were found in T (88.46%, Figure 2B_1_), in comparison to N (77.97%, Figure 2B_2_). Furthermore, a higher number of acetaminophen particles could be observed in T (27.61% more particles than in N), which was also expected due to the 20% higher acetaminophen content in comparison to N. Second, the sizes of acetylsalicylic acid particles were slightly smaller in N (76.71% < 2 µm; 19.18% 2–3 µm; 4.1% 4–5 µm, Figure 3A_2_) compared to T (61.6% < 2 µm; 29.46% 2–3 µm; 8.0% 4–5 µm, (Figure 3A_1_)), whereas for acetaminophen (Figure 3B_1_,B_2_) and for caffeine (Figure 3C_1_,C_2_), there were no significant differences observed between the two tablets. A subtle distinction in the agglomeration profile was visible. A higher incidence of bigger agglomerations could be observed in T for acetylsalicylic acid (Figure 5A_1_ vs. Figure 5A_2_).

Thus, Raman imaging allowed us to analyze chemical and spatial information from multiple APIs simultaneously, on a µm-scale, and to gain a first impression regarding the quality of the analyzed tablets in comparison to each other in a time-efficient way.

The presented method allows one to access relevant information for quality assurance of multiple active agents directly, non-invasively, and with high spatial resolution in a time efficient way. This means, that the technique could be applied online, and at different stages of development and manufacturing of multicomponent tablets. This could include for example the monitoring of the particle distribution before and after compaction, to understand the effect of different parameters of the pressing process on the API morphologies. For other settings, the FOV dimensions can be easily adjusted, simply by proper choice of the magnification of the objective lens (in contrast to ATR imaging where the FOV is given by the lens dimensions [63]). It could be also applied for monitoring (unwanted) crystallization processes at different physical conditions, e.g., during hot-melt extrusion processes, or for testing the effect of different storage conditions on the APIs in the formulation. Gaining thorough information about the API characteristics could facilitate efficient drug formulation development and could help avoid late stage quality failures and thus save resources.

## 3. Materials and Methods

### 3.1. Instrumentation of Fiber-Array-Based Raman Spectroscopic Imaging

**Figure 8:**
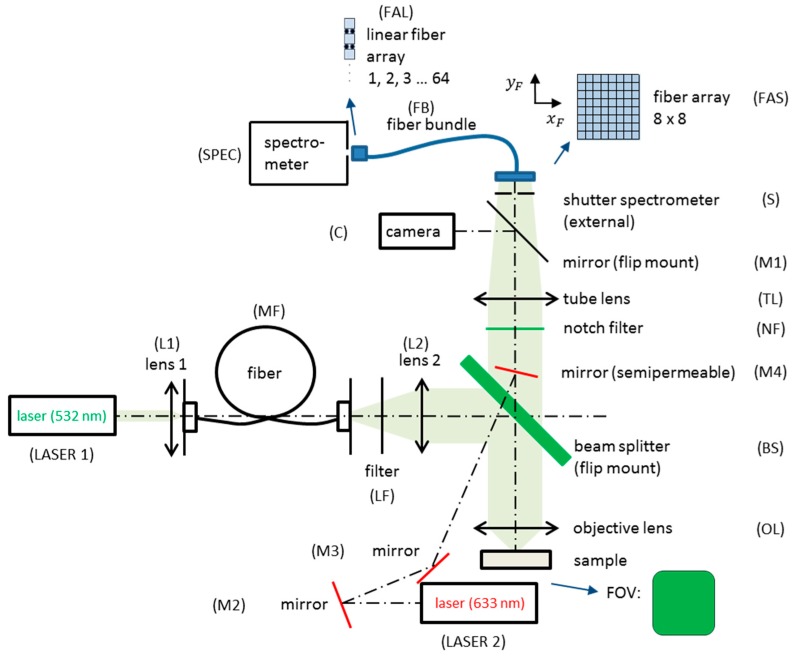
The experimental setup for fiber-array-based Raman hyperspectral imaging is divided by a beam splitter (BS) into an illumination and an imaging part. The Raman signals of the sample areas are collected with the help of a specially designed 8 × 8-fiber-array bundle (FB), transferred into a linear fiber-array (FAL) at the distal end, and positioned in the slit plane of the spectrometer (SPEC).

The experimental setup is the following (Figure 8): a laser with a wavelength of 532 nm (LASER1) is coupled by a lens (L1) into a step-index-multimode fiber (MF) with a squared core cross section to illuminate the sample homogeneously with a top hat intensity profile. The light from the fiber output passes through a lens (L2) and is reflected into an objective lens (OL) by a dichroitic beam splitter (BS). The objective lens focuses the light onto a square-shaped field of view (FOV) on the sample. It is used in combination with a tube lens. The FOV depends on the ratio of the focal lengths of lens 2 (L2) and the objective lens (OL). Within the scope of this study for a high spatial resolution, the most suitable magnification was 100×, resulting in a FOV area of 10 × 10 µm^2^ in the sample plane. The back-scattered light is collected by the objective lens (OL) with an NA of 0.9, defining the collection depth of about 1.6 µm with a 532 nm excitation wavelength. A notch filter (NF) reflects the laser excitation and Rayleigh-scattered light. Having passed the beam splitter (BS) and the tube lens (TL), the light can be directed by a flip mirror (M1) to a camera (C) to observe a microscopic image and choose an appropriate sample region. For the Raman spectroscopic measurements, the chosen sample region is imaged onto the entrance face of a fiber-array (FAS) with an 8 × 8-configuration. Sixty-four spectra can be collected simultaneously. The spatial resolution is 1.25 µm per image point. To realize a high fill factor, the cores of the fibers are square-shaped. The spectral information captured by the 8 × 8-fiber-array is transferred by a fiber bundle to the spectrometer (IsoPlane, Princeton Instruments) and the fibers are arranged in a line (FAL) in the plane of the entrance slit. A second laser with a wavelength of 633 nm (LASER 2) in combination with three mirrors (M2, M3, M4) are included in the setup. The 633 nm laser generates a spot with a diameter around 3 µm on the sample; it is used to identify the chosen sample area and for adjusting the fiber-array.

### 3.2. Measurement and Data Analysis

Two commercially available tablets were purchased from a local pharmacy: Thomapyrin Intensiv^®^ (tablet T) from Boehringer Ingelheim GmbH & Co. KG and Neuranidal^®^ N (tablet N) from STADA GmbH. Both tablets contain the same active ingredients: acetylsalicylic acid (aspirin), acetaminophen (paracetamol) and caffeine. For the full composition of the tablets please refer to the Supporting Information, Table S1. The coating of the tablets was carefully removed to ensure reliable detection of the APIs. The two commercial tablets T and N were used as easily-available samples with known API content and were suitable to demonstrate the applicability of the fiber-array imaging method to gain information about the API’s distribution, as well as the particle shape and size in pharmaceutical tablets with several APIs (such as the two investigated commercial tablet types) in one shot.

**Figure 9:**
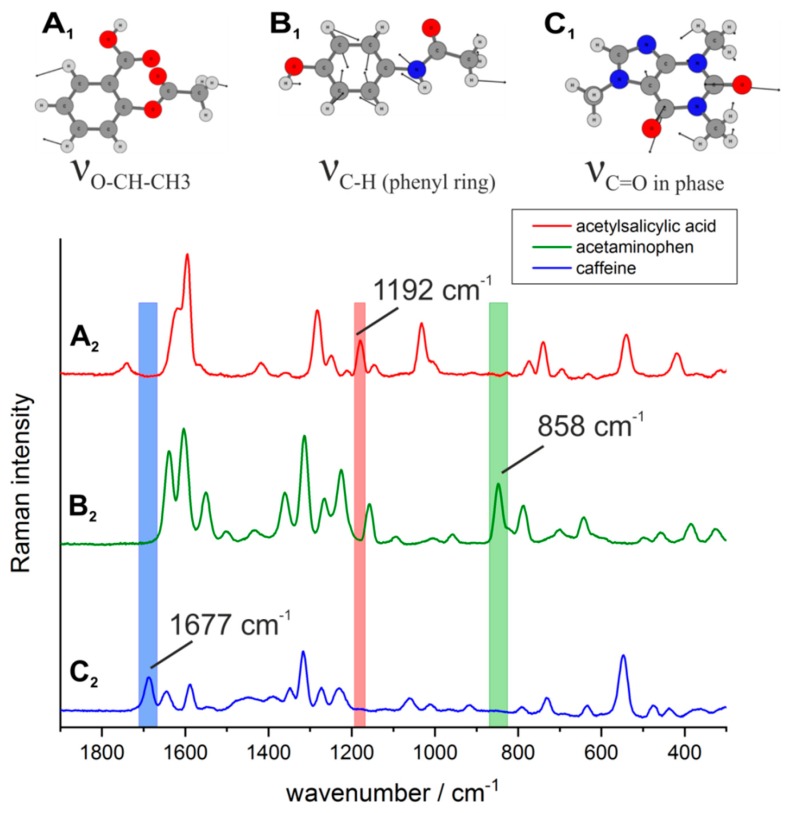
Characteristic vibrational modes and assignments of the active ingredients acetylsalicylic acid (**A_1_**, **A_2_**), acetaminophen (**B_1_**, **B_2_**) and caffeine (**C_1_**, **C_2_**).

One characteristic spectral band of each API was selected. The strong Raman bands at wavenumbers 1192 cm^−1^ for acetylsalicylic acid (ν_O-CH-CH3_-vibration), 858 cm^−1^ for acetaminophen (ν_C-H_-vibration) and 1677 cm^−1^ for caffeine (ν_C=O_ in phase-vibration) were proven the most suitable bands as they showed minimal spectral overlap among the APIs themselves (Figure 9) and with the excipients (Figure S1), respectively. In order to generate the hyperspectral images of the three APIs, the relative wavenumber range 835 to 873 cm^−1^ was used for acetaminophen, 1171 to 1202 cm^−1^ for acetylsalicylic acid and 1670 to 1705 cm^−1^ for caffeine (Figure 9). Vibrational modes depicted in Figure 9A_1_–C_1_ were calculated with the help of density functional theory (DFT) with Gaussian 09 [64]. The hybrid exchange-correlation functional with Becke’s three-parameter exchange functional (B3) [65] slightly modified by Stephens et al. [66] coupled with the correlation part of the functional from Lee, Yang, and Parr (B3LYP) [67] and Dunning’s triple (cc-pVTZ) correlation consistent basis sets of contracted Gaussian functions with polarized and diffuse functions [68] at standard conditions were applied. First, an intensity calibration was carried out on the fiber-array, using a piece of a silicon wafer as a homogeneous sample. The measurements of the tablets were carried out with an acquisition time of 5 s per fiber-array position and a laser power of 12 mW on the sample surface.

The intensity arrays corresponding to the different sample spots were superimposed and converted into color-coded images. In this study, the excipients were summed up as background.

A sample pattern consisting of squares with known dimensions (edge length of one square was 1 µm) acted as a size standard.

## 4. Conclusions

The potential of fiber-array based Raman spectroscopic imaging was presented as in-process control for rapid, chemically selective, and simultaneous analysis of particle shape, size and distribution of different active ingredients in tablets with high spatial resolution. Wide-field Raman imaging has several advantages in comparison to other elaborated chemical imaging techniques. Compared to mass-spectrometric imaging techniques, Raman imaging is performed in a non-destructive way and has high potential for cost efficient miniaturization. Spatial information is accessible down to 1 µm resolution, by using a high magnification.

The particle size, shape and distribution of the three active pharmaceutical ingredients, acetylsalicylic acid, acetaminophen and caffeine were analyzed in two different commercially available tablets, Thomapyrin^®^ Intensiv (T) and Neuranidal^®^ N (N). Different particle shapes were recognized for each API: needle-like and round ones for acetylsalicylic acid, as well as round particles for acetaminophen and caffeine. Different particle sizes were found for each API. The caffeine particles were the smallest ones. The distribution of the active ingredients was assessed in seven areas of the tablets respectively, presenting distinct patterns: in some areas clear particle shapes were recognized from all three APIs, whereas in other parts only acetylsalicylic acid and/or acetaminophen were dominant. Agglomerations of different dimensions of acetylsalicylic acid and acetaminophen could be clearly identified. Small differences were observed when comparing the two different commercially available tablets, T and N. A higher amount in acetaminophen was visible in T. More needle-shaped and bigger acetylsalicylic acid particles were found in the analyzed T tablets alongside a higher incidence of bigger agglomerations, in comparison to the N tablets. This gives an exemplary demonstration of the capabilities of the presented method.

Altogether, the potential of fiber-array-based Raman spectroscopic imaging was demonstrated for online quality control of pharmaceutical formulations, such as tablets.

## Supplementary Materials

The following are available online. Figure S1: Comparison of the Raman spectra of the active ingredients and the most important excipients in the tablets. Table S1: Composition of the commercial tablets T and N.
