# Supplementary material for: Fiber-Array-Based Raman Hyperspectral Imaging for Simultaneous, Chemically-Selective Monitoring of Particle Size and Shape of Active Ingredients in Analgesic Tablets
Source: Molecules. 2019 Nov 30;24(23):4381. doi: 10.3390/molecules24234381 (PMC6930444; doi:10.3390/molecules24234381)
Supplement: Supplementary file 1 [file molecules-24-04381-s001.pdf]

Supplementary Materials

Fiber-Array-Based Raman Hyperspectral Imaging for Simultaneous, Chemically-Selective Monitoring of Particle Size and Shape of Active Ingredients in Analgesic Tablets

Timea Frosch [1,†], Elisabeth Wyrwich [1,†], Di Yan [1], Juergen Popp [1,2,3] and Torsten Frosch [1,2,3,*]

[1] Leibniz Institute of Photonic Technology, 07745 Jena, Germany
[2] Institute of Physical Chemistry, Friedrich Schiller University, 07743 Jena, Germany
[3] Abbe Centre of Photonics, Friedrich Schiller University, 07745 code Jena, Germany
* Correspondence: torsten.frosch@uni-jena.de or torsten.frosch@gmx.de; Tel.: +49 3641 206 211
† These authors contributed equally to this work.

Table S1: Composition of the commercial tablets T and N.

	**Amount declared**	
**Tablet ingredient**	**Thomapyrin Intensiv® (T)**<br>**Weight: ~ 600 mg**	**Neuranidal® N (N)**<br>**Weight: ~ 650 mg**
Acetylsalicilic acid	250 mg	250 mg
Acetaminophen	250 mg	200 mg
Caffeine	50 mg	50 mg
Corn starch	✓	✓
Lactose monohydrate	✓	
Stearic acid	✓	✓
Talcum		✓

Corn starch and lactose monohydrate are filling excipients, whereas stearic acid and talcum are lubricants used on the tablet's surface for easier removal from the tableting stamp. It is expected that corn starch is used in highest amounts of the excipients, and talcum will be deposited on the outer surface of the tablet. Stearic acid is a saturated fatty acid, a wax-like substance, thus it is expected that it can penetrate deeper into the tablet than the first outer layer and it is generally used in very small amounts.

Therefore, corn starch and talcum are expected to contribute the second most after the active ingredients to the Raman signal of the tablet's surface and they were analyzed with the same set-up and measurement conditions as the active ingredients, see **Figure S1.**

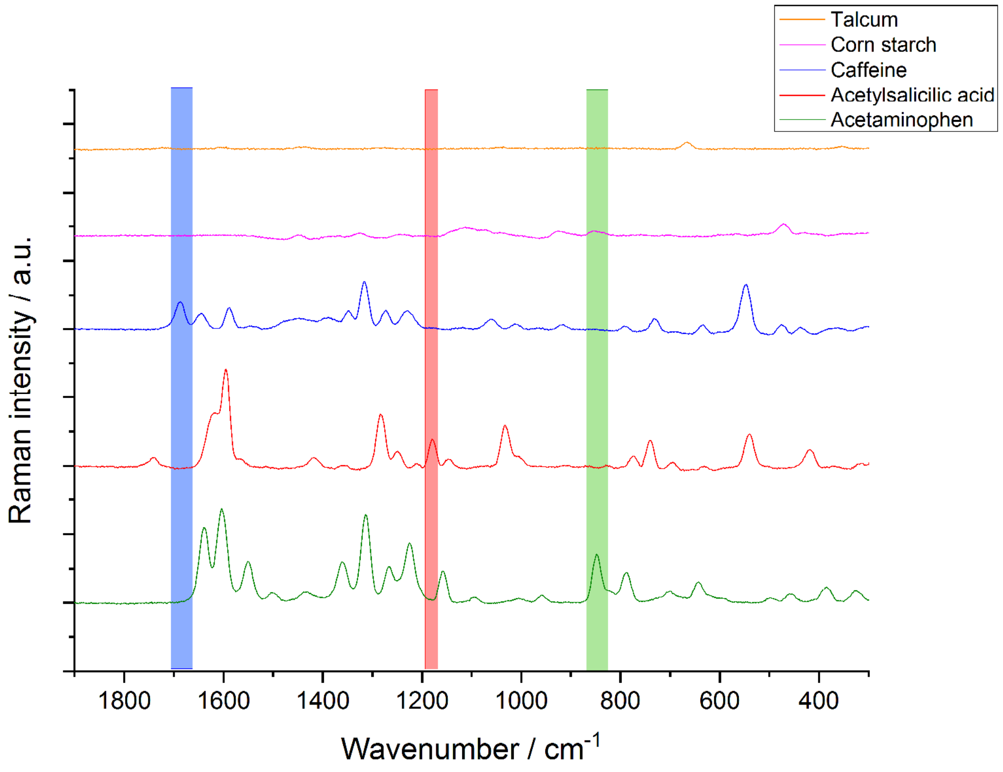

Figure S1: Comparison of the Raman spectra of the active ingredients acetaminophen (green), acetylsalicylic acid (red) and caffeine (blue) and the most important excipients corn starch (pink) and talcum (orange). The chosen characteristic vibrational modes for the identification of the active ingredients are highlighted.
